# Alternative objective function to predict reasonable muscle forces using a Hill-type muscle model

**DOI:** 10.1186/1757-1146-7-S1-A127

**Published:** 2014-04-08

**Authors:** Jongsang Son, Hoyoon Lee, Jongman Kim, Youngho Kim

**Affiliations:** 1 Department of Biomedical Engineering and Institute of Medical Engineering, Yonsei University, Wonju, 220-710, Republic of Korea

## Background

Joint moments modeled from a musculoskeletal tool differ from those recorded by a dynamometer. In order to solve the problem, numerical methods to minimize the variance of the joint moments have been adopted [1]. The existing objective function (EOF) in the optimization, however, might not be sufficient to estimate reasonable muscle forces due to a possibility of predicting well-matched joint moments with the combination of unrealistic individual muscle forces [2]. In this study, we introduce a new objective function (NOF) for predicting reasonable muscle forces and to compare its performance with EOF.

## Methods

NOF was designed to strengthen the linear relationship between: (1) the recorded and modeled joint moments, and (2) the muscle activations and the muscle forces. One male (age: 18 years; mass: 78 kg; height: 178 cm) participated in the study with the informed consent prior to commencing the experimental trials. Surface electrodes were attached to record EMG signals from elbow major muscles using an eight-channel surface EMG system (MyoSystem 1200, Noraxon Inc., USA). Dynamometer tasks were performed with Biodex System 3 Pro (Biodex Medical Systems, New York, USA) to measure elbow joint moments. The participant was asked to perform three maximum isometric contractions at 90° (flexed). The subject then generated an elbow flexion moment, rested, and generated an elbow extension moment. To evaluate the effects of NOF compared to EOF briefly, we focused on the changes in biceps brachii long head (BIClong) muscle force and compared the relative root-mean-square error.

## Results and discussion

**Figure 1:**
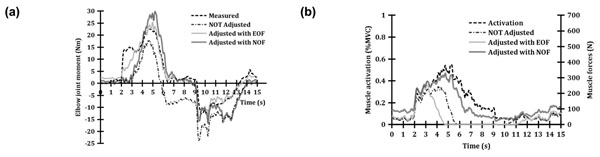
(a) Measured and predicted elbow joint moments, and (b) Muscle activation and force of long biceps brachii.

Modeled joint moments with no parameter calibration (NOT Adjusted) showed undesirable negative offset during the 3-second-rest period, but this problem was solved by the parameter calibration with EOF or NOF (Figure 1a). Even though EOF provided a good estimation of joint moment, it resulted from a combination of unrealistic muscle forces. BIClong muscle generated no force between about 5 s and 11 s despite of quite large muscle activity (Figure 1b). In contrast, the parameter calibration module with NOF predicted very similar muscle forces to the corresponding muscle activations. NOF predicted more desirable muscle forces than EOF, but the accuracy in predicting joint moments was relatively low. This might result from the fact that the number of possible value of model parameters with NOF are limited compared to EOF, because muscle forces to determine joint moments are constrained. This might be considered as a trade-off problem.

## Conclusions

Even though NOF yielded relatively low performance in joint moment prediction, it estimated muscle forces better, providing more reasonable kinetic information about human movements such as walking and running.
